# TV viewing during childhood and adult type 2 diabetes mellitus

**DOI:** 10.1038/s41598-021-83746-4

**Published:** 2021-03-04

**Authors:** Daniela Schmid, Walter C. Willett, Michele R. Forman, Ming Ding, Karin B. Michels

**Affiliations:** 1 Institute for Prevention and Cancer Epidemiology, Faculty of Medicine and Medical Center, University of Freiburg, Elsässerstr. 2, 79110 Freiburg, Germany; 2 Division for Quantitative Methods in Public Health and Health Services Research, Institute of Public Health, Medical Decision Making and Health Technology Assessment, UMIT-University for Health Sciences, Medical Informatics and Technology, Hall in Tyrol, Austria; 3 Department of Nutrition, Harvard T.H. Chan School of Public Health, Boston, MA USA; 4 Department of Epidemiology, Harvard T.H. Chan School of Public Health, Boston, MA USA; 5 Channing Division of Network Medicine, Department of Medicine, Brigham and Women’s Hospital, Harvard Medical School, Boston, MA USA; 6 Department of Nutrition Science, College of Health and Human Science, Purdue Center for Cancer Research, Purdue University, West Lafayette, IN USA; 7 Department for Epidemiology, Fielding School of Public Health, University of California, Los Angeles, CA USA

**Keywords:** Epidemiology, Outcomes research

## Abstract

We examined whether regular television (TV) viewing at ages 3–5 and 5–10 years is related to the incidence of type 2 diabetes mellitus (T2D) in adult women. We used data from 34,512 mother-nurse daughter dyads in the Nurses’ Health Study (NHS) II and the Nurses’ Mothers’ Cohort Study. Mothers of NHS II participants completed a questionnaire on their pregnancy with the nurse and her early life experience. During 391,442 person-years of follow-up from 2001 to 2013, 1515 nurses developed T2D. Increasing levels of TV viewing at 3–5 years of age retrospectively reported by the mothers were related to a greater risk of T2D in adulthood: multivariable-adjusted hazard ratios (HRs) for ≤ 1, 2, and ≥ 3 h/day vs. no TV viewing were 1.11 [95% confidence interval (CI) 0.96–1.28], 1.20 (95% CI 1.02–1.41), and 1.35 (95% CI 1.11–1.65), *p* trend = 0.002, respectively, after adjustment for early life variables, including childhood physical activity and adiposity. Retrospectively reported TV viewing for ≥ 3 h/day at 5–10 years of age was associated with a 34% greater risk of adult T2D (HR 1.34, 95% CI 1.05–1.70, *p* trend < 0.001). Additional adjustments for adult variables, including adult TV viewing and current BMI attenuated the effect estimates (≥ 3 h/day TV viewing at 3–5 years: HR 1.22, 95% CI 0.99–1.49, *p* trend = 0.07; TV viewing at 5–10 years: 1.16, 95% CI 0.91–1.49, *p* trend = 0.09). The present study suggests that TV viewing during early childhood increases risk of T2D in adult women; adult BMI explains part of this association. Further research is required to confirm this observation and understand the mediating pathways.

## Introduction

With 422 million people affected by diabetes worldwide, the past four decades have witnessed the quadrupling of diabetes incidence^1^. The proliferation in type 2 diabetes mellitus (T2D) is likely largely due to increases in overweight and obesity, and a lack of physical activity^2^. Sedentary behavior is recognized as a risk factor for major chronic diseases with the strongest and most persistent associations seen for T2D in adults^3,4^. Of particular concern is television (TV) viewing, one of the most prevalent sedentary behaviors among children, given its association with childhood obesity in both cross-sectional and longitudinal studies^5,6^. Children spend substantial time watching TV with national data indicating that 3- to 7-year-old children in the United States spend on average 2–3 h per day watching TV, with video and computer games adding an additional hour to children’s screen time^7^.

Although the association between TV viewing and the metabolic syndrome has been investigated in adolescence before^8^, no previous study has considered the role of TV viewing during childhood on the risk of T2D later in life. The perinatal and early life period is of particular importance since it is the phase of life marked by early adaptation in metabolic regulation and therefore represents a vulnerable time window for the development of T2D and heart disease in adult life^9,10^. It is also a time when children develop behaviors that may persist into later life.

To our knowledge, our study is the first to examine whether TV viewing at ages 3–5 years or 5–10 years is related to T2D in adulthood among women. We further estimated the magnitude of the associations explained by childhood body fat and body mass index (BMI) and TV viewing in adulthood using mediation models.

## Methods

### Study population

**Figure 1:**
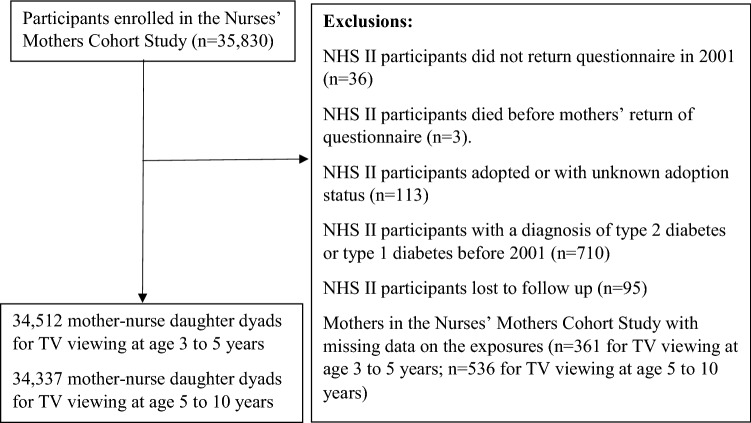
Flow chart of study selection process.

The NHS II cohort is an ongoing prospective cohort established in 1989 when 116,680 registered nurses aged between 25 and 42 years from 14 U.S. states completed a mailed baseline questionnaire on lifestyle factors and medical history^11^. Follow-up questionnaires were mailed to study members every two years updating information on lifestyle factors and health. In March 2001, 35,830 mothers of NHS II participants who were free of cancer participated in the Nurses’ Mothers Cohort Study and answered mailed questionnaires about their pregnancy and early live exposures of their daughters. Further details of the Nurses’ Mothers’ Cohort Study have been provided elsewhere^12^. We excluded NHS II participants who were adopted or whose adoption status is unknown, those with a diagnosis of type 2 before 2001, type 1 diabetes (T1D), and those with missing data on the exposures leaving 34,512 (TV viewing at age 3–5 years) and 34,337 (TV viewing at age 5–10 years) mother-nurse daughter dyads for the final analysis (Fig. 1). We compared baseline characteristics between participants included and excluded from our study (Supplemental Table S1). NHS II participants excluded from this study had a slightly higher family history of diabetes, marginally lower childhood and adult activity, and were slightly more likely to be overweight or obese during childhood and adulthood than those participants included, which was mainly due to our exclusion of NHS II participants with a diagnosis of diabetes before 2001, but were otherwise comparable (Supplemental Table S1).

The use of the data from the Nurses’ Health Study and the Nurses’ Mothers’ Cohort Study for this analysis was approved by the Nurses’ Health Study Working Group. The study protocol was approved by the institutional review boards of the Brigham and Women’s Hospital and Harvard School of Public Health, Boston. Responding to our questionnaires was considered implied consent.

### Assessment of childhood TV viewing

In the Nurses’ Mothers’ Cohort questionnaire, nurses’ mothers reported the hours their daughters’ spent watching TV at the ages of 3–5 years. The following question was asked: “Between the ages of 3 and 5 years, how many hours per day during the week did your nurse daughter watch TV?”. Response categories were “No TV,” “up to 0.5 h/day,” “ about 1 h/day,” “about 2 h/day,” “about 3 h/day,” “4 h/day,” and “5 or more hours/day.” We collapsed the categories into four categories to ensure a sufficient number of participants across groups: none, > 0 ≤ 1 h, 2 h, and ≥ 3 h/day. The question is based on a similar question in the Behavioral Risk Behavior Survey for Youth: CDC with self-reported response which was moderately correlated with self-reported weekly viewing diaries (r = 0.50) and demonstrated fair test–retest reliability (r = 0.30) when the same question was completed one week apart^13,14^. The Mothers Cohort questionnaire also inquired about hours watching TV per day at the ages of 5–10 years; upper categories were combined in a similar manner. To assess potential windows of vulnerability, these exposures were analyzed separately.

### Covariate information

Mothers of the nurses provided information on maternal, perinatal and early life risk factors of their daughters including: maternal education, pre-pregnancy weight, height, weight gain during pregnancy, smoking during pregnancy, and physical activity during pregnancy; paternal weight and height; nurse daughter’s gestation age at birth, birthweight, and breast feeding. Mothers further provided information about physical activity of their daughters between the ages of 3–5 and 5–10 years by answering the following question at both ages: “How would you best describe your nurse daughter’s activity level compared to other girls of similar age?” Response categories were: “highly physically active,” “active,” “mostly inactive,” and “inactive.” In the NHS II baseline questionnaire, nurses provided information on their body fatness at ages 5 and 10 years by choosing one of nine pictorial body diagrams (somatotypes) developed by Stunkard et al.^15^ that best depicted their body fatness with levels ranging from 1 (represents the most lean) and 9 (represents the most overweight). Somatotypes were combined into five categories. The validity of this measure of body shape in early life have been assessed among 181 participants aged 71–76 years in the Third Harvard Growth Study^16^. Information on current age of the nurses, race, family history of diabetes, was collected from the NHS II baseline questionnaire. We used biennially updated information about caloric intake, alcohol intake, smoking, physical activity, TV viewing, and BMI during adulthood from the NHS II questionnaires.

### Ascertainment of T2D

In each biennial questionnaire, nurses were asked whether they had been diagnosed with T2D in the past 2 years. Respondents reporting a diagnosis were then mailed a supplemental questionnaire about the results of diagnostic tests, symptoms, and therapy. The diagnosis was confirmed if at least one of the following was reported according to the National Diabetes Data Group criteria^17^: (1) one or more classic symptoms (excessive thirst, polyuria, weight loss, hunger) and fasting plasma glucose concentrations ≥ 7.8 mmol/L or random plasma glucose concentrations ≥ 11.1 mmol/L; (2) two or more elevated plasma glucose concentrations on different occasions (fasting concentrations ≥ 7.8 mmol/L, random plasma glucose concentrations ≥ 11.1 mmol/L, and/or concentrations of ≥ 11.1 mmol/L after ≥ 2 h shown by oral-glucose-tolerance testing) in the absence of symptoms; or (3) treatment with hypoglycemic medication (insulin or oral hypoglycemic agent).

The American Diabetes Association diagnostic criteria for T2D diagnosis were used from 1998 onward using the fasting glucose concentration cutoff of 7.0 mmol/L. In a validation study, about 98% cases were reconfirmed against blinded physician medical record review^18^.

### Statistical analysis

Cox proportional hazards regression models were computed conditioned on age and follow-up cycle to estimate hazard ratios (HRs) and 95% confidence intervals (CIs) of T2D across increasing categories of TV viewing at ages 3–5 years or 5–10 years. To allow for a prospective analysis of T2D risk, participants contributed follow-up time from the return of the NHS II questionnaire in 2001 until their last returned questionnaire, the date of death, T2D diagnosis, or end of follow-up on June 1, 2013. Participants who experienced a competing risk event were censored from further analyses. Women were also censored at the date of type 1 diabetes mellitus (T1D).

We tested the proportional hazards assumption by plotting a graph on the log negative log survival against log of survival time. The proportional hazards assumption was considered satisfied since largely parallel lines between different groups of exposure were found (Supplemental Figure S1). In an additional test of the proportional hazards assumption we examined separate models during the first 6 years (2001–2007) and during the second 6 years of follow-up (2007–2013); we also ran models that included interaction terms between TV viewing during childhood and time period. The associations with T2D were similar during the two time periods and no significant interaction was observed.

Multivariable-adjusted models were adjusted for perinatal variables, adiposity during childhood, and adult variables of the nurses including physical activity, TV viewing,current BMI, caloric intake, alcohol consumption, and smoking. Adult variables were updated biannually from the NHS II follow-up questionnaires and treated as time-varying covariates in the analytic model. The fully adjusted model had maternal education (1–3 years of high school or less, 4 years of high school, 1–3 years of college, 4 + years of college), maternal pre-pregnancy BMI (quintiles), paternal BMI at nurses’ birth (quintiles), maternal weight gain during pregnancy (< 10 lbs, 10–14 lbs, 15–19 lbs, 20–29 lbs, 30–40 lbs, ≥ 40 lbs), maternal smoking during pregnancy [no smoking during pregnancy, quit smoking during pregnancy, light smoker (< 15 cigarettes/day), heavy smoker (≥ 15 cigarettes/day)], maternal physical activity during pregnancy (occupational, household, and recreational physical activity combined into one variable: mostly inactive/mostly sit or stand, mostly walking/active, highly active/heavy lab or housework), gestation age (< 38 weeks, 38–42 weeks, > 42 weeks), birth weight (< 2500 g, 2500–2999 g, 3000–3499 g, 3500–3999, ≥ 4000 g), breast feeding (no or < 1 week, 1 week-3 months, 3–6 months, 6–9 months, 6–12 months), body fatness at ages 5 or 10 years (five groups), physical activity at 3–5 years or 5–10 years (inactive, mostly inactive, active, highly active), race (White, Non-White), family history of diabetes (yes, no), age of the nurses (continuous), BMI (< 20.9 kg/m^2^, 21–22.9 kg/m^2^, 23–24.9 kg/m^2^, 25–29.9 kg/m^2^, 30–34.9 kg/m^2^, + 35 kg/m^2^), caloric intake (quintiles), alcohol intake (quintiles), smoking (never, past, current < 15 cigarettes/d, current ≥ 15 cigarettes/d), physical activity (quintiles), and TV viewing (none, 1–10 h/week, 11–20 h/week, 21–40 + hours/week) during adulthood. If covariate values were missing the previous value was either carried forward for one time interval or a missing indicator category was created.

We calculated the proportion^19^ of the association between TV viewing during childhood and diabetes later in life that was mediated by adiposity during childhood, adult BMI, and TV viewing using the publicly available % Mediate macro (http://www.hsph.harvard.edu/donna-spiegelman/software/mediate/). In addition, we evaluated joint associations of TV viewing during childhood and TV viewing during adulthood with adult T2D risk. To test for a linear trend across categories of exposures, we created a continuous variable using the midpoint of each category. All statistical analyses were performed using the SAS statistical software version 9.4 (SAS Institute Inc., Cary, NC).

## Results

During 391,442 person-years of follow-up, 1515 newly diagnosed cases of T2D occurred between 2001 and 2013.The distribution of early life and adult variables of the nurses across categories of TV viewing at ages 3–5 years and 5–10 years is shown in Table 1 (variables values are averages during follow-up). Nurses whose mothers retrospectively reported that they spent more time watching TV during childhood were more likely to currently smoke and to spend greater time in front of the TV in adulthood and had a somewhat higher adult BMI than those whose mothers reported that they were less likely watching TV in early life. Moreover, increasing duration of TV viewing in childhood was more likely related to maternal smoking during pregnancy and lower parental education (Table 1).

**Table 1:** Age-standardized characteristics of daughters of the Nurses’ Mothers’ Cohort according to television viewing at ages 3–5 and 5–10 years. Values are averages during follow-up.

	Childhood TV viewing at ages 3–5 years				Childhood TV viewing at ages 5–10 years			
	None	> 0 to 1 h/d	2 h/d	≥ 3 h/d	None	> 0 to 1 h/d	2 h/d	≥ 3 h/d
Age	51.3 (5.9)	50.8 (5.6)	50.8 (5.6)	50.7 (5.6)	51.2 (5.9)	51.0 (5.8)	50.9 (5.7)	50.8 (5.7)
Race, white, %	94.7	97.2	97.2	95.3	93.1	97.0	96.9	96.1
Family history of diabetes, %	23.2	21.9	23.4	25.7	21.5	21.4	23.8	26.0
*Perinatal/early life variables*								
Maternal pre-pregnancy BMI, kg/m^2^	21.3 (2.7)	21.2 (2.5)	21.3 (2.7)	21.5 (2.8)	21.4 (2.7)	21.1 (2.5)	21.3 (2.7)	21.4 (2.7)
Paternal BMI at the nurse’s birth, kg/m^2^	23.4 (2.7)	23.6 (2.8)	23.7 (2.9)	23.7 (2.9)	23.3 (2.7)	23.5 (2.7)	23.6 (2.8)	23.7 (2.9)
Maternal physical activity during pregnancy (highly active), %	3.5	1.9	1.6	1.9	3.9	2.3	1.8	1.7
Maternal weight gain during pregnancy, lb	41.0	42.4	42.9	41.8	41.3	41.9	42.7	41.9
Maternal education, 1 to + 4 yrs of college; %	41.0	41.4	31.3	22.6	42.6	43.8	33.2	23.8
Paternal education, 1 to + 4 yrs of college; %	44.8	48.5	39.2	30.4	45.9	50.4	39.9	31.7
Childhood activity (highly active), %	34.5	34.5	31.3	29.1	31.1	32.0	28.1	26.4
Maternal smoking during pregnancy, %	14.4	21.1	22.4	25.6	10.3	19.2	22.1	25.2
Gestation age, wks	39.4 (2.3)	39.4(2.3)	39.4(2.3)	39.4(2.3)	39.4(2.2)	39.4(2.3)	39.4(2.3)	39.4(2.3)
Birth weight, g	3311 (522)	3288 (507)	3293 (501)	3281 (514)	3336 (519)	3292 (511)	3290(505)	3284(513)
Breast feeding, %	61.1	51.6	48.5	47.6	65.7	53.6	49.8	50.0
Overweight/obese during childhood (somatotype ≥ 5), %	6.0	5.7	6.4	8.3	10.8	9.7	11.2	13.7
*Adult variables*								
Caloric intake, kcal	1848 (562)	1829 (553)	1812 (552)	1812 (562)	1864 (573)	1833 (550)	1817 (556)	1814 (560)
Alcohol intake, g/d, mean	5.6 (9.5)	6.5 (10.1)	6.4 (10.3)	5.9 (10.1)	4.7 (9.2)	6.4 (9.9)	6.3 (10.1)	6.0 (10.3)
Current smoking, %	5.8	7.5	8.5	9.2	5.1	6.7	8.4	9.5
Physical activity, MET-h/wk	22.4 (27.8)	24.1(28.3)	23.3 (29.5)	21.4 (29.5)	21.9 (28.6)	24.2 (27.9)	23.0 (28.8)	21.3 (28.8)
TV viewing, hrs/wk	8.7 (8.1)	8.8 (9.0)	9.6 (9.7)	10.3 (10.3)	7.3 (7.2)	8.5 (8.6)	9.6 (9.4)	10.5 (10.7)
BMI, kg/m^2^	26.7 (6.0)	26.4(5.8)	26.9(6.0)	27.5 (6.3)	26.4 (5.8)	26.3 (5.8)	26.8 (6.0)	27.6 (6.4)

Values are means (SD) or percentages and are standardized to the age distribution of the study population (except age).

*TV* television, *BMI *body mass index.

### TV viewing at ages 3–5 years

In the age-adjusted model, 3 or more hours of daily TV viewing at ages 3–5 years were associated with greater risk of adult T2D compared with no TV viewing (HR 1.56, 95% CI 1.28–1.90, *p* trend < 0.001, Table 2). The following variables were added to the multivariable-adjusted models, e.g., perinatal variables (HR 1.36, 95% CI 1.11–1.66, *p* trend = 0.001), body fatness at age 5 years (HR 1.35, 95% CI 1.11–1.65, *p* trend = 0.002), adult life variables (HR 1.30, 95% CI 1.07–1.59, *p* trend = 0.01), adult TV viewing (HR 1.26, 95% CI 1.03–1.54, *p* trend = 0.02), and adult BMI (HR 1.22, 95% CI 0.99–1.49, *p* trend = 0.07); and the positive association between TV viewing at ages 3–5 years and later T2D risk was attenuated with their inclusion (Table 2).

**Table 2:** Hazard ratios and 95% confidence intervals for adult diabetes incidence by category of television viewing at ages 3–5 years among 34,512 participants of the Nurses’ Health II cohort between 2001 and 2013.

	TV viewing at ages 3–5 years				
	None	> 0 to 1 h/d	2 h/d	≥ 3 h/d	*P* trend
*Cases/Person-years*	363/83,175	616/177,077	369/95,168	167/36,022	
Model 1: Age-adjusted	1.00	1.07 (0.93–1.23)	1.24 (1.06–1.45)	1.56 (1.28–1.90)	< 0.001
Model 2: Multivariable-adjusted	1.00	1.11 (0.96–1.28)	1.20 (1.02–1.41)	1.36 (1.11–1.66)	0.001
Model 3: Model 2- + childhood body shape-adjusted	1.00	1.11 (0.96–1.28)	1.20 (1.02–141)	1.35 (1.11–1.65)	0.002
Model 4: Model 3- + adult variables-adjusted	1.00	1.13 (0.98–1.30)	1.19 (1.01–1.40)	1.30 (1.07–1.59)	0.01
Model 5: Model 4 + adult TV viewing-adjusted	1.00	1.12 (0.97–1.30)	1.17 (0.99–1.37)	1.26 (1.03–1.54)	0.02
Model 6: Model 5 + adult BMI-adjusted	1.00	1.14 (0.99–1.32)	1.14 (0.97–1.34)	1.22 (0.99–1.49)	0.07

*TV* television.

Model 1: adjusted for age of nurse (continuous) and follow-up cycle.

Model 2: same as model 1 but with additional adjustment for race (White, Non-White), family history of diabetes (yes, no), maternal education (1–3 yrs of high school or less, 4 yrs of high school, 1–3 yrs of college, 4 + yrs of college), maternal pre-pregnancy body mass index (quintiles), paternal body mass index at nurses’ birth (quintiles), maternal weight gain during pregnancy (< 10 lbs, 10–14 lbs, 15–19 lbs, 20–29 lbs, 30–40 lbs, ≥ 40 lbs), maternal smoking during pregnancy [no smoking during pregnancy, quit smoking during pregnancy, light smoker (< 15 cigarettes/day), heavy smoker (≥ 15 cigarettes/day)], maternal physical activity during pregnancy (occupational, household, and recreational physical activity combined, mostly inactive/mostly sit or stand, mostly walking/active, highly active/heavy lab or housework), gestational age (< 38 wks, 38–42 wks, > 42 wks), birth weight (< 2500 g, 2500-2999 g, 3000-3499 g, 3500–3999, ≥ 4000 g), breast feeding (No or < 1 wk, 1 wk-3 months, 3–6 months, 6–9 months, 6–12 months), and physical activity at 3–5 years (inactive, mostly inactive, active, highly active).

Model 3: same as model 2 but with additional adjustment for body shape at 3–5 years (five groups).

Model 4: same as model 3 but with additional adjustment for adult caloric intake (quintiles), adult alcohol intake (quintiles), adult smoking (never, past, current < 15 cigarettes/d, current ≥ 15 cigarettes/d), and adult physical activity (quintiles).

Model 5: Adult TV viewing (0–3.5 h/wk, 3.5–7 h/wk, 7–14 h/wk, 14–21 h/wk, + 21–40 h/wk).

Model 6: same as model 5 but additional adjustment for adult body mass index (< 20.9 kg/m^2^, 21–22.9 kg/m^2^, 23–24.9 kg/m^2^, 25–29.9 kg/m^2^, 30–34.9 kg/m^2^, + 35 kg/m^2^).

Based on mediation analysis, adult BMI and TV viewing were responsible for 25.3% (p < 0.001) and 12.5% (*p* < 0.001) of the positive association of TV viewing at ages 3–5 years with adult T2D incidence, respectively. Body fatness at age 3–5 years appears not to be a mediator of this relation (proportion of the childhood TV viewing effect explained by childhood body adiposity < 1%).

### TV viewing at ages 5–10 years

Long duration of TV viewing (≥ 3 h/day) versus no TV viewing at ages 5–10 years was related to a greater risk of T2D in adult life (age-adjusted HR 1.58, 95% CI 1.25–1.99, *p* trend < 0.001, Table 3). Further adjustment for perinatal variables, including physical activity at ages 5–10 years and childhood adiposity (HR 1.34, 95% CI 1.05–1.70, *p* trend < 0.001), as well as adult life factors (HR 1.32, 95% CI 1.04–1.68, *p* trend = 0.002) revealed attenuated but positive associations between TV viewing at ages 5–10 years and later T2D risk. The associations were further attenuated and no longer significant following additional adjustment for adult TV viewing (HR 1.26; 95% CI 0.99–1.60, *p* trend = 0.01) and BMI (HR 1.16; 95% CI 0.91–1.49, *p* trend = 0.09) although a trend for a dose–response relation remained evident. Adult BMI and TV viewing were estimated to mediate 36.1% (*p* < 0.001) and 15.3% (*p* < 0.001) of this association, respectively, whereas childhood adiposity appears not to mediate this association (*p* = 0.13).

**Table 3:** Hazard ratios and 95% confidence intervals for adult diabetes incidence by category of television viewing at the ages 5–10 years among 34,431 participants of the Nurses’ Health II cohort between 2001 and 2013.

	TV viewing at ages 5–10 years				
	None	> 0 to 1 h/d	2 h/d	≥ 3 h/d	*P* trend
*Cases/Person-years*	102/25,617	562/167,235	567/139,809	267/56,824	
Model 1: Age-adjusted	1.00	1.02 (0.82–1.26)	1.30 (1.04–1.61)	1.58 (1.25–1.99)	< 0.001
Model 2: Multivariable-adjusted	1.00	1.05 (0.84–1.30)	1.23 (0.98–1.53)	1.36 (1.07–1.73)	< 0.001
Model 3: Model 2- + childhood body shape-adjusted	1.00	1.04 (0.83–1.29)	1.21 (0.97–1.51)	1.34 (1.05–1.70)	< 0.001
Model 4: Model 3- + adult variables-adjusted	1.00	1.09 (0.88–1.36)	1.24 (0.99–1.54)	1.32 (1.04–1.68)	0.002
Model 5: Model 4- + adult TV viewing-adjusted	1.00	1.07 (0.86–1.33)	1.19 (0.95–1.48)	1.26 (0.99–1.60)	0.01
Model 6: Model 5 + adult BMI-adjusted	1.00	1.07 (0.86–1.33)	1.19 (0.95–1.49)	1.16 (0.91–1.49)	0.09

*TV* television.

Model 1: adjusted for age of nurse (continuous) and follow-up cycle.

Model 2: same as model 1 but with additional adjustment for race (White, Non-White), family history of diabetes (yes, no), maternal education (1–3 yrs of high school or less', 4 yrs of high school, 1–3 yrs of college, 4 + yrs of college), maternal pre-pregnancy body mass index (quintiles), paternal body mass index at nurses’ birth (quintiles), maternal weight gain during pregnancy (< 10 lbs, 10–14 lbs, 15–19 lbs, 20–29 lbs, 30–40 lbs, ≥ 40 lbs), maternal smoking during pregnancy [no smoking during pregnancy, quit smoking during pregnancy, light smoker (< 15 cigarettes/day), heavy smoker (≥ 15 cigarettes/day)], maternal physical activity during pregnancy (occupational, household, and recreational physical activity combined, mostly inactive/mostly sit or stand, mostly walking/active, highly active/heavy lab or housework), gestational age (< 38 wks, 38–42 wks, > 42 wks ), birth weight (< 2500 g, 2500–2999 g, 3000–3499 g, 3500–3999, ≥ 4000 g), breast feeding (No or < 1 wk, 1 wk-3 months, 3–6 months, 6–9 months, 6–12 months), and physical activity at 3–5 years (inactive, mostly inactive, active, highly active).

Model 3: same as model 2 but with additional adjustment for body shape at 5–10 years (five groups).

Model 4: same as model 3 but with additional adjustment for adult caloric intake (quintiles), adult alcohol intake (quintiles), adult smoking (never, past, current < 15 cigarettes/d, current ≥ 15 cigarettes/d),and adult physical activity (quintiles).

Model 5: Adult TV viewing (0–3.5 h/wk, 3.5–7 h/wk, 7–14 h/wk, 14–21 h/wk, + 21–40 h/wk).

Model 6: same as model 5 but additional adjustment for adult body mass index (< 20.9 kg/m^2^, 21–22.9 kg/m^2^, 23–24.9 kg/m^2^, 25–29.9 kg/m^2^, 30–34.9 kg/m^2^, + 35 kg/m^2^).

### Joint associations of childhood TV and adult TV viewing

In an analysis of the joint associations of childhood and adult TV viewing with risk of T2D later in life, the combinations of 2 h/day of TV viewing at ages 3–5 years and > 1 to 2 h/day of TV viewing during adulthood or regular TV viewing at ages 3–5 years (≥ 3 h/day) and during adulthood were related to a greater risk of adult T2D compared to ≤ 1 h/day of childhood TV viewing and adult TV viewing, although these associations were attenuated following additional adjustment for current BMI (Table 4). By comparison, high volumes of TV viewing at ages 3–5 years (2 or ≥ 3 h/day) and low TV viewing during adulthood (≤ 1 h/day) were related to a non-significant greater risk of adult T2D. Similar observations were made for the combinations of TV viewing at ages 5–10 years and TV viewing during adulthood with later T2D risk (Table 5).

**Table 4:** Joint associations of TV viewing at ages 3–5 years and during adulthood with diabetes in adult life among participants of the Nurses’ Health II cohort between 2001 and 2013.

	Childhood TV viewing		
Adult TV viewing	Age 3–5 years		
	≤ 1 h/d	2 h/d	≥ 3 h/d
** ≤ 1 h/d**			
Cases	196	81	32
Multivariable-adjusted HR (95% CI)	1.00	1.24 (0.95–1.62)	1.33 (0.91–1.95)
Multivariable- + adult BMI-adjusted HR (95% CI)	1.00	1.12 (0.86–1.47)	1.26 ( 0.85–1.85)
**> 1 to 2 h/d**			
Cases	174	66	30
Multivariable-adjusted HR (95% CI)	1.45 (1.18–1.79)	1.55 (1.17–2.06)	1.72 (1.16–2.54)
Multivariable- + adult BMI-adjusted HR (95% CI)	1.26 (1.02–1.55)	1.26 (0.94–1.68)	1.46 (0.98–2.17)
**≥ 3 h/d**			
Cases	252	89	53
Multivariable-adjusted HR (95% CI)	1.77 (1.46–2.15)	1.76 (1.36–2.28)	2.13 (1.55–2.92)
Multivariable- + adult BMI-adjusted HR (95% CI)	1.33 (1.09–1.61)	1.33 (1.03–1.73)	1.52 (1.10–2.09)

*TV* television, *HR *hazard ratio, *CI *confidence interval.

Multivariable model adjusted for age of nurse (continuous) and follow-up cycle, race (White, Non-White), family history of diabetes (yes, no), maternal education (1–3 yrs of high school or less', 4 yrs of high school, 1–3 yrs of college, 4 + yrs of college), maternal pre-pregnancy body mass index (quintiles), paternal body mass index at nurses’ birth (quintiles), maternal weight gain during pregnancy (< 10 lbs, 10–14 lbs, 15–19 lbs, 20–29 lbs, 30–40 lbs, ≥ 40 lbs), maternal smoking during pregnancy [no smoking during pregnancy, quit smoking during pregnancy, light smoker (< 15 cigarettes/day), heavy smoker (≥ 15 cigarettes/day)], maternal physical activity during pregnancy (occupational, household, and recreational physical activity combined, mostly inactive/mostly sit or stand, mostly walking/active, highly active/heavy lab or housework), gestational age (< 38 wks, 38–42 wks, > 42 wks ), birth weight (< 2500 g, 2500-2999 g, 3000-3499 g, 3500–3999, ≥ 4000 g), breast feeding (No or < 1 wk, 1 wk-3 months, 3–6 months, 6–9 months, 6–12 months), and physical activity at 3–5 years (inactive, mostly inactive, active, highly active), body shape at 3–5 years (5 groups), adult caloric intake (quintiles), adult alcohol intake (quintiles), adult smoking (never, past, current < 15 cigarettes/d, current ≥ 15 cigarettes/d), and adult physical activity (quintiles).

**Table 5:** Joint associations of TV viewing at ages 5–10 years and during adulthood with diabetes in adult life among participants of the Nurses’ Health II cohort between 2001 and 2013.

	Childhood TV viewing		
Adult TV viewing	Age 5–10 years		
	≤ 1 h/d	2 h/d	≥ 3 h/d
** ≤ 1 h/d**			
Cases	140	101	65
Multivariable-adjusted HR (95% CI)	1.00	1.20 (0.92–1.55)	1.78 (1.32–2.42)
Multivariable- + adult BMI-adjusted HR (95% CI)	1.00	1.19 ( 0.91–1.55)	1.57 (1.15–2.13)
** > 1 to 2 h/d**			
Cases	125	100	43
Multivariable-adjusted HR (95% CI)	1.57 (1.23–2.01)	1.68 (1.29–2.19)	1.66 (1.17–2.36)
Multivariable- + adult BMI-adjusted HR (95% CI)	1.37 (1.07–1.76)	1.44 (1.11–1.88)	1.34 (0.94–1.92)
** ≥ 3 h/d**			
Cases	161	152	76
Multivariable-adjusted HR (95% CI)	1.86 (1.47–2.34)	2.01 (1.58–2.54)	2.08 (1.56–2.78)
Multivariable- + adult BMI-adjusted HR (95% CI)	1.39 (1.10–1.76)	1.56 (1.23–1.98)	1.49 ( 1.11–1.99)

*TV* television, *HR *hazard ratio, *CI* confidence interval.

Multivariable model adjusted for age of nurse (continuous) and follow-up cycle, race (White, Non-White), family history of diabetes (yes, no), maternal education (1–3 yrs of high school or less', 4 yrs of high school, 1–3 yrs of college, 4 + yrs of college), maternal pre-pregnancy body mass index (quintiles), paternal body mass index at nurses’ birth (quintiles), maternal weight gain during pregnancy (< 10 lbs, 10–14 lbs, 15–19 lbs, 20–29 lbs, 30–40 lbs, ≥ 40 lbs), maternal smoking during pregnancy [no smoking during pregnancy, quit smoking during pregnancy, light smoker (< 15 cigarettes/day), heavy smoker (≥ 15 cigarettes/day)], maternal physical activity during pregnancy (occupational, household, and recreational physical activity combined, mostly inactive/mostly sit or stand, mostly walking/active, highly active/heavy lab or housework), gestational age (< 38 wks, 38–42 wks, > 42 wks ), birth weight (< 2500 g, 2500-2999 g, 3000-3499 g, 3500–3999, ≥ 4000 g), breast feeding (No or < 1 wk, 1 wk-3 months, 3–6 months, 6–9 months, 6–12 months), and physical activity at 5–10 years (inactive, mostly inactive, active, highly active), body shape at 5–10 years (5 groups), adult caloric intake (quintiles), adult alcohol intake (quintiles), adult smoking (never, past, current < 15 cigarettes/d, current ≥ 15 cigarettes/d), and adult physical activity (quintiles).

## Discussion

In this study, TV viewing in early and later childhood as retrospectively reported by mothers of participants was positively associated with risk of T2D later in life. The association was independent of childhood physical activity and childhood adiposity but appears to be partially explained by adult BMI.

To the best of our knowledge, no previous study examined the relation of TV viewing during childhood to the risk for T2D later in life. In four previous meta-analyses, a positive association between TV viewing during adulthood and diabetes risk had a summary risk that ranged from 1.20 to 2.19^3,4,20,21^.

Biologic mechanisms to explain these observations are unclear but may involve pathways related to energy expenditure. Spending hours at a time watching TV can displace time spent being physically active^22^ and higher caloric intake associated with TV viewing^23^ can lead to a positive energy balance, thereby promoting obesity and obesity-related metabolic disruption (e.g., reduced insulin sensitivity), and the risk for diabetes. Other proposed mechanisms relating watching TV to dysmetabolic effects may include impaired sleep quality^24^, distraction from habitual food intake control or satiety, and a reduced number of family meals consumed^25^. Health behaviors acquired in childhood may lead to persistent behavioral and biological adaptations contributing to greater vulnerability to chronic diseases in adulthood.

previous longitudinal study^8^ using data from the Northern Swedish Cohort reported that watching 'several shows a day' compared with 'one show/week' or less at age 16 years was related to the metabolic syndrome at age 43 years following adjustment for TV viewing at ages 21 and 30 years and other potential confounders (OR 1.86, 95% CI 1.06–3.27), but the association was attenuated after adding BMI at age 30 to the model. Although the current literature suggests a link between sedentary behavior and adiposity among children^26^, the role of BMI in the relation between sedentary behavior and disease outcomes is not clear yet. Our results indicate a relation of TV viewing in early childhood with T2D in adulthood that is independent of adiposity during childhood; however, additional control for adult variables, in particular adult BMI attenuated this association. Adjusting for adult BMI, however, may not provide the most informative analytic model since adult BMI is an intermediate variable. More research is required to confirm these observations and clarify the mediating pathways.

Adult metabolic diseases may originate in childhood, but studying such associations in a longitudinal study is challenging since several decades of follow-up would be necessary. Assessing TV watching in childhood through mothers’ reports makes an unbiased study feasible: while the frequency of TV watching during childhood was retrospectively assessed, prevalent cases of T2D among the daughters were excluded at baseline. Hence, at the time of reporting of their daughters’ childhood behavior mother had no knowledge whether their daughter might develop T2D later excluding the chance of recall bias; any error in the retrospective reporting of TV watching by the mothers would be random in nature. Only incidence cases of T2D diagnosed during the subsequent 12 years were included in our analyses. Therefore, we were able to embed the retrospective assessment of the exposure in a prospective follow-up.

Our study has both strengths and limitations. One strength of our study includes its two-generational design, which provides the unique opportunity to examine data collected directly from the mothers and combine them with data independently provided by the nurses. Further strengths of our study are its prospective nature, the large number of study participants, and adjustment for numerous potential confounding variables in early and adult life. Despite our study pointing towards a positive association between TV viewing during childhood and adult T2D, our findings need to be interpreted in the context of certain limitations. Although, we carefully controlled for numerous potential confounding variables in our models, unmeasured confounding or residual confounding may have occurred. Moreover, exposure assessment is based on maternal recall decades later and lack of memory may have led to some degree of non-differential misclassification in our study. The Nurses’ Mothers’ Cohort questionnaire has not directly been validated for the exposures examined herein, but the questions are similar to assessments of TV viewing during adulthood in the NHS II questionnaire reported to have reasonable validity^27^. While the long recall period for the exposures by maternal proxy reports is a challenge, differential misclassification was avoided by our prospective design which included only incident cases occurring after 2001. Further, parents may be the only reliable source of TV watching habits of their children and since TVs were still relatively new during the exposure period examined the reports are likely to have a good validity. Another limitation is that our study population is > 95% Caucasian white. Black and Hispanic children have been reported to watch significantly more TV, have higher rates of overweight and obesity, higher consumption of sugar-sweetened beverages, but lower rates of maternal smoking during pregnancy than non-HispanicWhite, or Asian children^28–30^, limiting the generalizability of our findings.

The proportion of childhood TV viewing does not represent the longer screen time among children today, which is typical for prospective cohort studies. By 1950, nine percent of American households had a television set, a proportion that had increased to 64.5 percent only five years later and reached 93% in 1955^31^. Current screen time is a lot more heterogeneous and is not limited to TV viewing only. Thus, our associations in all likelihood may underestimate the influence of extended screen time patterns in current adolescents on their future metabolic health suggesting an even more pressing need to establish intervention programs.

Our study identified TV viewing during childhood as a potential risk factor for T2D later in life. This observation supports recommendations to limit TV viewing during childhood to one hour per day as advocated by the American Academy of Pediatrics^32^. More research is needed to confirm our findings and to build the evidence base for precise sedentary behavior guidelines beyond broad recommendations to reduce sedentary behavior.

## Conclusion

Our study is the first investigation on childhood TV viewing and risk of developing T2D later in life suggesting that long hours spent watching TV during childhood may increase risk for T2D in adulthood. Given that frequent TV viewing at an early age may predict long-term behavior pattern and physiologic adaptation during life, promoting reduction in screen time and improvement of physical activity beginning in early age may be crucial to reduce the long-term risk for diabetes. Future studies are needed to continue this line of evidence.

## Supplementary Information

Supplementary Information

## Abbreviations

TV: Regular television
T2D: Type 2 diabetes mellitus
HR: Hazard ratio
CI: Confidence interval
NHS: Nurses’ Health Study
BMI: Body mass index
