# Supplementary material for: TV viewing during childhood and adult type 2 diabetes mellitus
Source: Sci Rep. 2021 Mar 4;11:5157. doi: 10.1038/s41598-021-83746-4 (PMC7933176; doi:10.1038/s41598-021-83746-4)

TV Viewing During Childhood and Adult Type 2 Diabetes Mellitus

Daniela Schmid, Ph.D.,[1,2] Walter C. Willett, M.D., Dr. P.H,[3,4,5] Michele R. Forman, Ph.D. ,[6] Ming Ding, Sc.D.,[3] Karin B. Michels, Sc.D, Ph.D. [1,7]

Affiliations:

[1]Institute for Prevention and Cancer Epidemiology, Faculty of Medicine and Medical Center, University of Freiburg, Freiburg, Germany
[2]Division for Quantitative Methods in Public Health and Health Services Research, Institute of Public Health, Medical Decision Making and Health Technology Assessment, UMIT-University for Health Sciences, Medical Informatics and Technology, Hall in Tyrol, Austria
[3]Department of Nutrition, Harvard T.H. Chan School of Public Health, Boston, MA, USA
[4]Department of Epidemiology, Harvard T.H. Chan School of Public Health, Boston, MA, USA
[5]Channing Division of Network Medicine, Department of Medicine, Brigham and Women's Hospital, Harvard Medical School, Boston, MA, USA
[6]Department of Nutrition Science, College of Health and Human Science, Purdue Center for Cancer Research, Purdue University, West Lafayette, IN, USA
[7]Department for Epidemiology, Fielding School of Public Health, University of California, Los Angeles, CA, USA

Address correspondence to:
Karin B. Michels, ScD, PhD
Institute for Prevention and Cancer Epidemiology
University of Freiburg, Faculty of Medicine and Medical Center
Elsässerstr. 2
79110 Freiburg
Germany
Email: karin.michels@uniklinik-freiburg.de
Phone +49 761 270-77360
Fax +49 761 270-77340

Supplemental Table 1. Baseline characteristics between participants included and excluded from our study.

	Childhood TV viewing at ages 3 to 5		Childhood TV viewing at ages 5 to 10	
	Participants included in our study (n=34512)	Participants excluded from our study (n=1318)	Participants included in our study (n=34337)	Participants excluded from our study (n=1493)
Age	46.0 (4.6)	47.1 (4.5)	46.0 (4.6)	47.1 (4.6)
Race, white, %	96.5	95.0	96.5	95.3
Family history of diabetes, %	23.4	33.1	23.4	31.7
Perinatal/early life variables				
Maternal pre-pregnancy BMI, kg/m²	21.3 (2.6)	21.7 (3.1)	21.3 (2.6)	21.5 (3.0)
Paternal BMI at the nurse's birth, kg/m²	23.6 (2.8)	23.9 (3.1)	23.6 (2.8)	23.8 (3.1)
Maternal physical activity during pregnancy (highly active), %	2.1	2.3	2.1	2.1
Maternal weight gain during pregnancy, lb	42.0	38.3	42.0	39.3
Maternal education, 1 to +4 yrs of college; %	36.7	32.4	36.7	32.8
Paternal education, 1 to +4 yrs of college; %	43.3	38.3	43.2	40.0
Childhood activity (highly active), %	33.2	28.4	29.6	21.9
Maternal smoking during pregnancy, %	20.7	21.1	20.7	21.4
Gestation age, wks	39.4 (2.3)	39.3 (2.4)	39.4 (2.3)	39.3 (2.4)
Birth weight, g	3290.3 (510.2)	3234.5 (547.0)	3290.5 (510.5)	3236.2 (530.1)
Breast feeding, %	53.0	51.2	53.0	49.8
Overweight/obese during childhood (somatotype ≥5), %	6.3	9.6	11.1	16.2
Adult variables				

Caloric intake, kcal	1853 (552)	1830 (547)	1852 (552)	1848 (562)
Alcohol intake, g/d, mean	5.7 (9.4)	4.2 (8.6)	5.7 (9.4)	4.2 (7.9)
Current smoking, %	9.1	10.7	9.1	10.1
Physical activity, MET-h/wk	21.3 (26.3)	17.3 (24.6)	21.3 (26.3)	18.6 (26.6)
TV viewing, hrs/wk	0.3 (0.5)	0.3 (0.5)	0.3 (0.5)	0.4 (0.5)
BMI, kg/m²	26.2 (5.8)	30.3 (8.5)	26.3 (6.0)	30.0 (8.6)

Values are means (SD) or percentages and are standardized to the age distribution of the study population (except age). TV, television; BMI, body mass index

Supplemental Figure. Log negative log survival against the log of time (in years).

a. Age 3-5 years

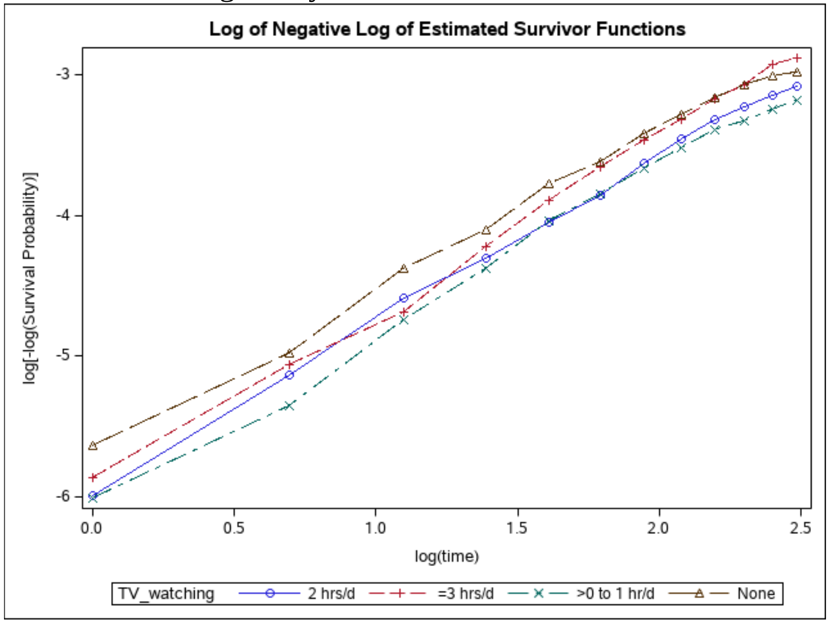

b. Age 5-10 years

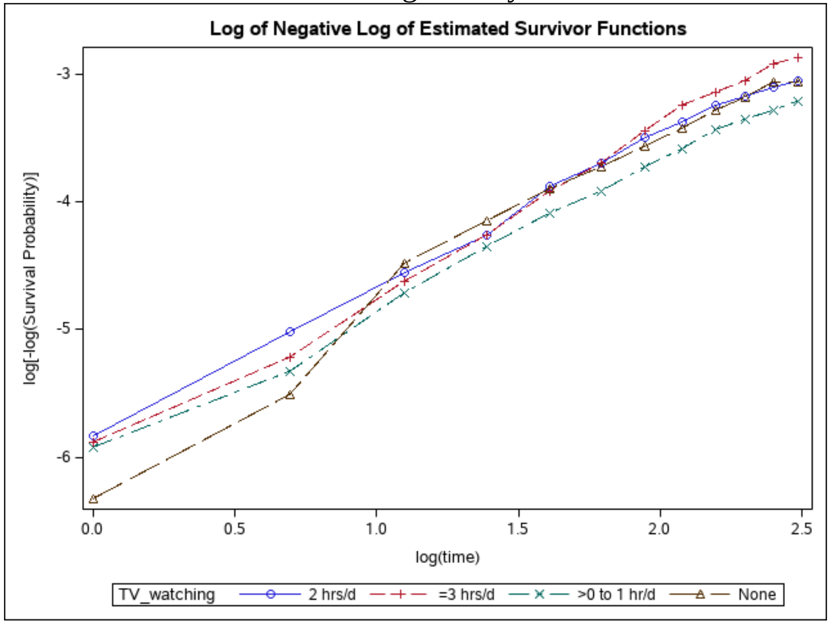

Supplement: Supplementary file 1 — Supplementary Information [file 41598_2021_83746_MOESM1_ESM.pdf]
